# Petersen hernia after open gastrectomy with Roux-en-Y reconstruction: a report of two cases and literature review

**DOI:** 10.1186/s40064-015-1556-8

**Published:** 2015-12-02

**Authors:** Akira Baba, Shinji Yamazoe, Murat Dogru, Yumi Okuyama, Takuji Mogami, Yuko Kobashi, Yosuke Nozawa, Yutaka Aoyagi, Hiroto Fujisaki, Masaharu Ogura, Junichi Matsui

**Affiliations:** Department of Radiology, Tokyo Dental College Ichikawa General Hospital, 5-11-13, Sugano, Ichikawa, Chiba 2728513 Japan; Department of Surgery, Tokyo Dental College Ichikawa General Hospital, 5-11-13, Sugano, Ichikawa, Chiba 2728513 Japan

**Keywords:** Petersen hernia, Internal hernia, Roux-en-Y reconstruction, Gastric cancer

## Abstract

Petersen hernia is a rare internal hernia that occurs after Roux-en-Y (R-Y) reconstruction. To our knowledge, there are a few reports on internal hernia, especially Petersen hernia after open gastrectomy for gastric cancer. Two rare cases of Petersen hernia are presented in this report. A man in his 70s was referred to our hospital due to a complaint of postprandial sudden abdominal pain. He had a history of open total gastrectomy with R-Y jejunal reconstruction through the antecolic route for gastric corpus cancer. On computed tomography (CT), bowel obstruction and strangulation of the small intestine were suspected. Emergency laparotomy was done, and an internal herniation of the small intestine through Petersen space was observed. A man in his 50s was referred to our hospital due to a complaint of severe sudden abdominal pain. He had a history of open gastrectomy and abdominal/lower intrathoracic esophageal resection with R-Y jejunal reconstruction of an antecolic jejunal limb for esophagogastric junction carcinoma. On CT, internal herniation of the small intestine was suspected. During emergency laparotomy, an internal herniation of the bowel through the Petersen space was observed. Though history of R-Y reconstruction surgery may be helpful, preoperative diagnosis of Petersen hernia is difficult to establish. Here we present two rare cases of this type of internal hernia.

## Background

In 1900, Dr. Walther Petersen, a German surgeon, first described Petersen space hernia which is an internal hernia caused by the Petersen defect, a space between the Roux limb and the transverse mesocolon formed after Roux-en Y (R-Y) reconstruction (Petersen 1900). This is a rare internal hernia that occurs after any type of gastrojejunostomy. Internal hernias after laparoscopic gastrectomy with R-Y reconstruction in bariatric surgery have been reported frequently, but reports about these after open surgery for gastric cancer are a few. To our knowledge, reports on internal hernia, especially Petersen hernia, after open gastrectomy for gastric cancer treated with R-Y reconstruction are a few. We report two cases of Petersen hernia after laparotomy for gastric cancer treated with R-Y reconstruction.

## Case description

### Case 1

A man in his 70s was referred to our emergency department due to postprandial sudden abdominal pain. Laboratory investigations revealed nonspecific findings, with a slight elevation of the inflammatory status (CRP 2.06 mg/L; WBC 9700/μl; AST 43 U/l; LDH 263 U/l; γ-GTP 76 U/l; AMY 535 IU/L). He had a history of open total gastrectomy with R-Y reconstruction with an antecolic jejunal limb for gastric corpus cancer a year prior to his presentation at our hospital and coronary bypass operation. The body mass index (BMI) was 19.1 kg/m^2^ at the time of surgery for Petersen hernia and 20.1 kg/m^2^ at the first gastrectomy.

**Fig. 1:**
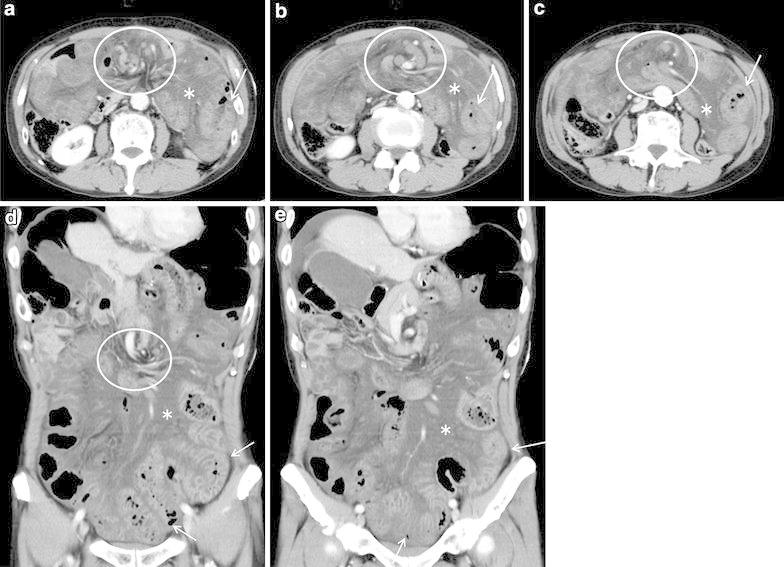
**a**–**c** Contrast-enhanced abdominal CT (90 s after injection of contrast agent) axial image of case 1; **d**, **e** contrast-enhanced abdominal CT (90 s after injection of contrast agent) coronal image of case 1. The dilated small intestine with a thickened bowel wall (*arrow*) with a high density area of mesenteric fat presenting with edematous changes (*asterisk*). Strangulation of the small intestine and mesenteric fat, and vascular structures presenting as the whirl sign (*circle*) can also be identified

**Fig. 2:**
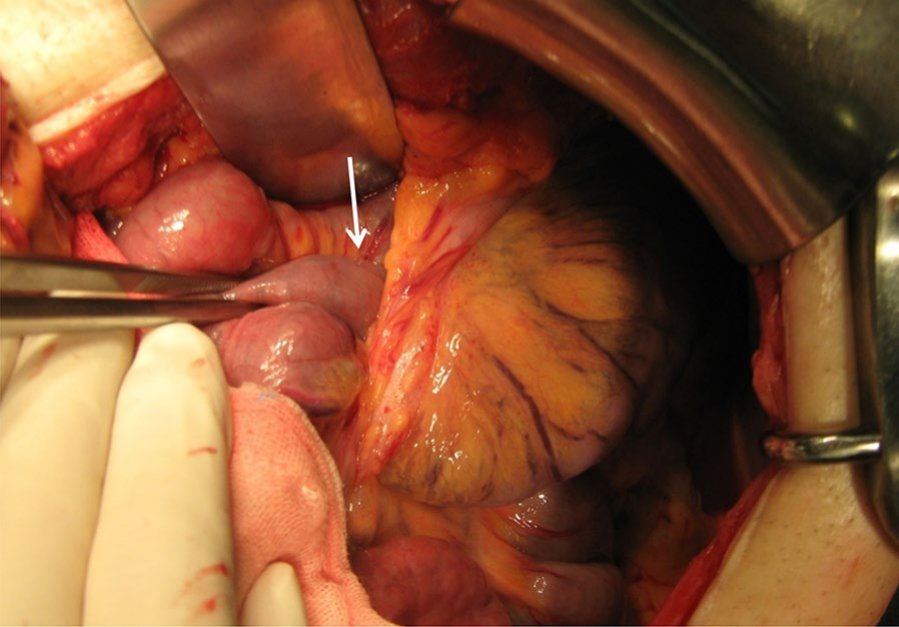
Intraoperative image of case 1. The small bowel herniating through the Petersen defect (*arrow*)

Other medical and family histories were unremarkable. Abdominal contrast enhanced computed tomography (CT) revealed a dilated small intestine with thickened bowel wall, high density of mesenteric fat presenting with edematous changes, and spiraling and converging mesenteric fat and vascular structure, showing “whirl sign”. Accordingly, bowel obstruction and strangulation of the small intestine were suspected (Fig. 1). Differential diagnoses included adhesive obstruction, internal herniation, and/or gastrointestinal infection. Emergency laparotomy showed an internal herniation of the small intestine through the Petersen space formed by transverse mesocolon and Roux jejunal limb from the left to the right side (Fig. 2). The herniated small intestine had more than one rotation. There was no color change suggesting the possibility of extensive bowel necrosis. The bowel obstruction was relieved, and the defect was closed by suturing. Since the operation, the patient was followed up regularly. No recurrence of bowel obstruction was observed for almost 2 years. However, the patient presented with adhesive bowel obstruction three times since then, and every time, the symptoms were relieved conservatively.

### Case 2

**Fig. 3:**
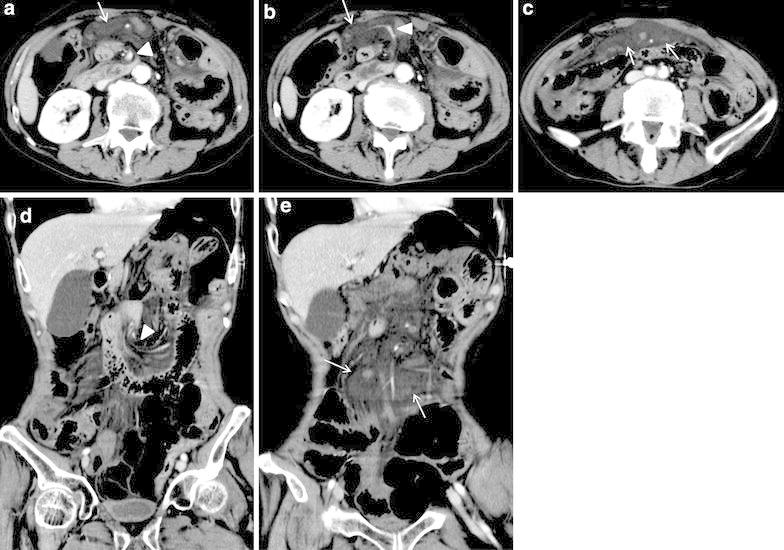
**a**–**c** Contrast-enhanced abdominal CT (90 s after injection of contrast agent) axial image of case 2; **d**, **e** contrast-enhanced abdominal CT (90 s after injection of contrast agent) coronal image of case 2. The localized high intensity of mesenteric fat (*arrow*) and strangulated SMA presenting with a whirl sign (*arrow head*)

**Fig. 4:**
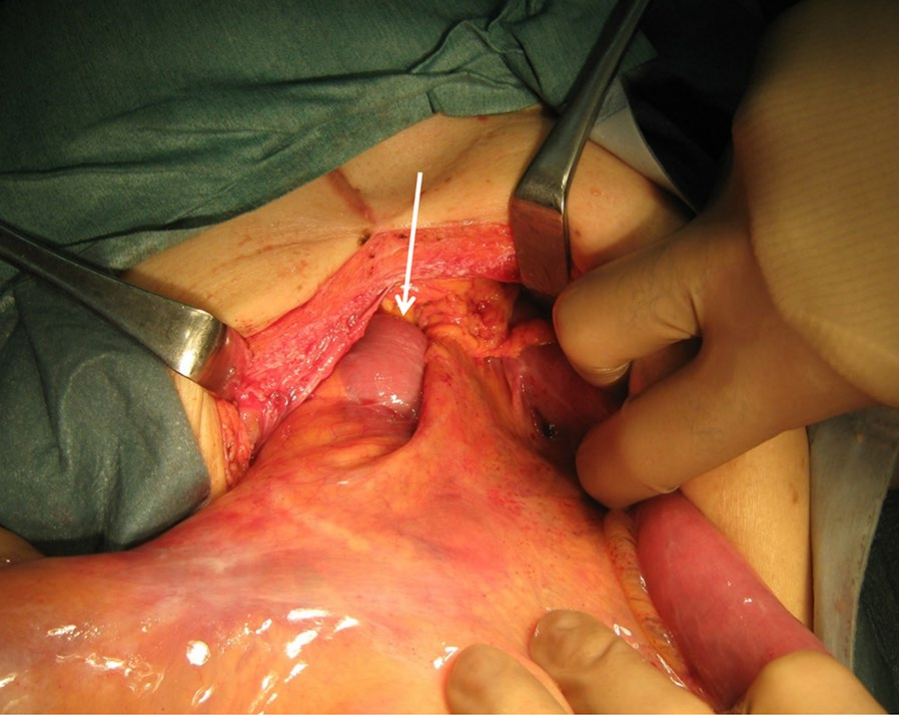
Intraoperative image of case 2. The small bowel herniating through the Petersen defect (*arrow*)

A man in his 50s was referred to our emergency department due to a sudden onset of severe abdominal pain. Laboratory investigations revealed non-specific findings (CRP 9.07 mg/L; AMY 154 IU/L). He had a history of open total gastrectomy and resection of abdominal/lower intrathoracic esophagus with R-Y jejunal reconstruction through antecolic route for gastroesophageal junction carcinoma a year and a half prior to the presentation. He had been followed up for outpatient visits and received TS-1. The BMI was 21.7 kg/m^2^ at the time of surgery for Petersen hernia, and 23.2 kg/m^2^ at the first gastrectomy. Medical and family histories were unremarkable, except for right leg amputation. On abdominal contrast enhanced CT, well-circumscribed high density of mesenteric fat, and strangulation of superior mesenteric artery (SMA) and vein (SMV) were present with the whirl sign, indicating internal herniation (Fig. 3). Emergent laparotomy was carried out, and internal hernia of the small intestine through Petersen space from left to right was discovered (Fig. 4). Prominent mesenteric edema was observed without color change, suggesting small bowel necrosis. The obstruction of intestine was relieved, and the defect was closed by suturing. The patient was followed up and there has been no recurrence of intestinal obstruction for 2.5 years.

## Discussion and Evaluation

Internal hernia is a well-known complication of R-Y gastric bypass. In laparotomy, the incidence has been reported to be 1–5 % (Paroz et al. 2006), and slightly higher in laparoscopic surgery (3.1–9.7 %) (Paroz et al. 2006; Higa et al. 2003; Capella et al. 2006). Several reports indicate that laparotomy may lead to more adhesion, which possibly prevents bowel loops internal herniation (Kojima et al. 2014; Lockhart et al. 2007; Miyagaki et al. 2012). In fact, open gastrectomy bears less risk of internal hernia than laparoscopic gastrectomy.

Body weight loss is considered to be another risk factor for internal hernia after gastrectomy (Kojima et al. 2014; Miyagaki et al. 2012). Weight loss after gastrectomy is due to a decrease in mesenteric fat (Kojima et al. 2014; Miyagaki et al. 2012). A greater loss of weight can induce an increase in the size of mesenteric or Petersen defect, thus increasing the risk of internal hernia (Kojima et al. 2014; Miyagaki et al. 2012). In our study both cases showed decreases in body weight and BMI after the surgeries. Male gender is also considered to be a risk factor of internal herniation in gastrectomy with R-Y reconstruction (Yoshikawa et al. 2014).

Another factor to consider is the position of Roux limb. R-Y reconstruction has become more popular in recent years, with the intention to avoid anastomotic leakage and reflux inflammation of the remnant stomach. Recently, many reports prefer an antecolic figure for the Roux limb, which reduces the potential hernia sites from three to two, including Petersen defect after laparoscopic R-Y bypass reconstruction (Kojima et al. 2014). Antecolic position may be reasonable because of reduction of the mesocolon defect, which is one of the most common sites of herniation (Kojima et al. 2014). In contrast, retrocolic position is known to have a higher risk of internal herniation (Yoshikawa et al. 2014; Reiss and Garg 2014). Some reports even indicate a high probability of strangulated obstruction and massive bowel resection with the retrocolic procedure (Yoshikawa et al. 2014).

In a recent report, however, all patients with Petersen hernia had undergone antecolic R-Y reconstructions (Yoshikawa et al. 2014). This report suggests that antecolic reconstruction procedure may tend to specifically lead to Petersen hernia (Yoshikawa et al. 2014).

**Fig. 5:**
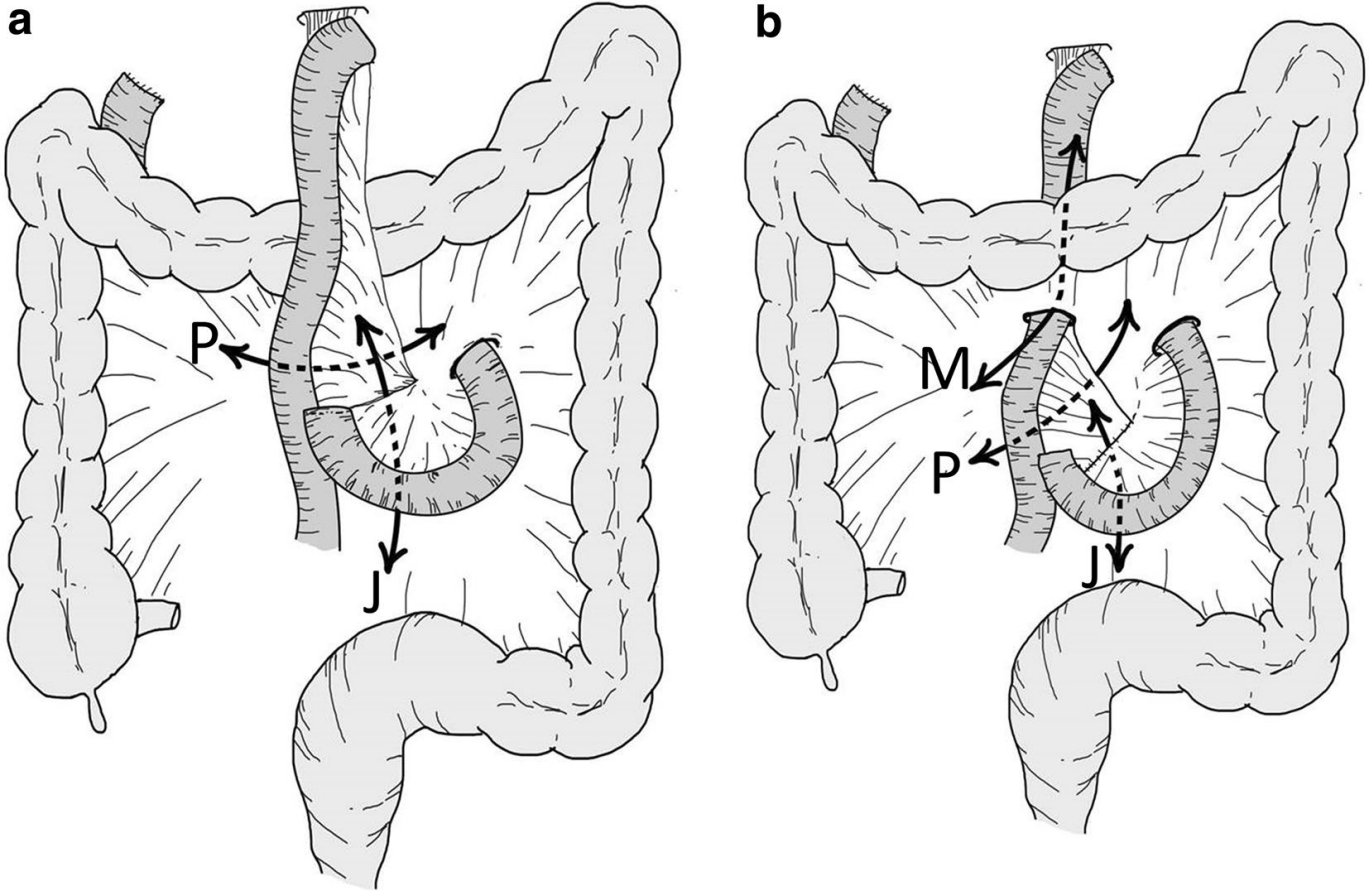
Scheme of the R-Y reconstruction. **a** Antecolic route; **b** retrocolic route. The mesenteric defect at the jejunojejunostomy (J), and the Petersen defect (P) can be seen in both the antecolic and the retrocolic routes. The defect in the transverse mesocolon through which the Roux loop passes is formed in the retrocolic route (M)

Petersen hernia is a specific type of hernia where the small bowel moves into the potential space between the caudal surface of the transverse mesocolon and the edge of the Roux limb. Petersen hernia is rarer than other internal hernias that have mesocolon mesentery defects and jejunojejunostomy after R-Y reconstruction (Garza et al. 2004). R-Y reconstruction procedure forms two (Fig. 5a) or three herniation sites (Fig. 5b). There are a few reports on internal hernia, especially Petersen hernia, after open gastrectomy for cancer treated with R-Y reconstruction. Petersen hernia can rapidly lead to acute bowel obstruction with necrosis, and when diagnosed, emergency surgery should be performed (Kojima et al. 2014; Yoshikawa et al. 2014). Delayed intervention may result in intestinal necrosis with the need for resection of variable lengths of the small bowel, with resultant morbidity and possible mortality (Yoshikawa et al. 2014).

As the symptoms of internal hernia, including Petersen hernia, are nonspecific, making its diagnosis is challenging. Most patients complain nonspecific symptoms such as postprandial abdominal pain, nausea, and emesis (Lockhart et al. 2007). Some patients may have recurrent transient herniation and intermittent abdominal pain (Reiss and Garg 2014). Furthermore, laboratory and radiographic findings may be normal (Srikanth et al. 2004).

Though not specific, several CT findings are suggestive of internal hernia; including the whirl sign, target sign, small bowl obstruction, clustered loop, retraction of the mesentery, congestion of mesenteric fat and vessels, mushroom sign, hurricane eye, small bowel behind SMA, and right-sided anastomosis (Lockhart et al. 2007; Reiss and Garg 2014; Garza et al. 2004; Blachar and Federle 2002; Gunabushanam et al. 2009; Fan et al. 2008; Onopchenko 2005). The CT anatomy in the patients who have undergone laparoscopic R-Y reconstruction is complicated, making imaging diagnosis difficult for internal hernia (Lockhart et al. 2007). While the CT findings are not almost always definite for the diagnosis of internal hernia, the whirl sign of mesenteric fat or vessels has been reported to be the best single predictor of hernia, with a sensitivity of approximately 80 % and specificity of 90 % (Lockhart et al. 2007). In previous reports, CT with oral and intravenous contrast has been reported to help clarify these findings and enhance the accuracy of diagnosis (Reiss and Garg 2014). Occasionally, CT may show no definite evidence of abnormality suggesting bowel obstruction (Srikanth et al. 2004; Onopchenko 2005). We do recommend that these CT findings should be assessed by radiologists who should also pay attention to the patient’s history of R-Y reconstruction (Lockhart et al. 2007).

In conclusion, we reported two rare cases of Petersen hernia after laparotomy with R-Y jejunal bypass reconstruction in this study. Petersen hernia presenting as internal hernia after R-Y reconstruction is rare, and its preoperative diagnosis is difficult to establish. Neither symptoms nor CT finding alone cannot lead to definitive diagnosis of Petersen hernia. Therefore, we should pay attention to any history of R-Y reconstruction and consult radiologist as needed in order to make a correct diagnosis and avoid massive bowel resection.

## Abbreviations

R-Y: Roux-en-Y
CT: computed tomography
CRP: C-reactive protein
WBC: white blood cell count
AST: aspartate transaminase
LDH: lactate dehydrogenase
γ-GTP: γ-glutamyl trans peptidase
AMY: amylase
BMI: body mass index
SMA: superior mesenteric artery
SMV: superior mesenteric vein
